# scMicrobe PTA: near complete genomes from single bacterial cells

**DOI:** 10.1093/ismeco/ycae085

**Published:** 2024-07-12

**Authors:** Robert M Bowers, Veronica Gonzalez-Pena, Kartika Wardhani, Danielle Goudeau, Matthew James Blow, Daniel Udwary, David Klein, Albert C Vill, Ilana L Brito, Tanja Woyke, Rex R Malmstrom, Charles Gawad

**Affiliations:** DOE Joint Genome Institute, Lawrence Berkeley National Laboratory, Berkeley, CA, United States; Department of Pediatrics, Stanford University, Stanford, CA, United States; Department of Pediatrics, Stanford University, Stanford, CA, United States; DOE Joint Genome Institute, Lawrence Berkeley National Laboratory, Berkeley, CA, United States; DOE Joint Genome Institute, Lawrence Berkeley National Laboratory, Berkeley, CA, United States; DOE Joint Genome Institute, Lawrence Berkeley National Laboratory, Berkeley, CA, United States; Department of Pediatrics, Stanford University, Stanford, CA, United States; Chan Zuckerberg Biohub, San Francisco, CA, United States; Chan Zuckerberg Biohub, San Francisco, CA, United States; DOE Joint Genome Institute, Lawrence Berkeley National Laboratory, Berkeley, CA, United States; DOE Joint Genome Institute, Lawrence Berkeley National Laboratory, Berkeley, CA, United States; Department of Pediatrics, Stanford University, Stanford, CA, United States; Chan Zuckerberg Biohub, San Francisco, CA, United States

**Keywords:** single cell genomics, unculturable bacteria, microbiome, microbial ecology, primary template-directed amplification, multiple displacement amplification, whole genome amplification, microbiome sequencing, mobile genetic elements, biosynthetic gene clusters

## Abstract

Microbial genomes produced by standard single-cell amplification methods are largely incomplete. Here, we show that primary template-directed amplification (PTA), a novel single-cell amplification technique, generated nearly complete genomes from three bacterial isolate species. Furthermore, taxonomically diverse genomes recovered from aquatic and soil microbiomes using PTA had a median completeness of 81%, whereas genomes from standard multiple displacement amplification-based approaches were usually <30% complete. PTA-derived genomes also included more associated viruses and biosynthetic gene clusters.

## Introduction

Difficulties in cultivating most bacterial and archaeal species presents a barrier to exploring the genetic make-up of the Earth's microbiomes. To access the genomes of most microorganisms, culture-independent methods such as shotgun metagenomic sequencing [1–3] and single-cell sequencing [4–8] can be employed. While metagenomics has led to unprecedented insights into the metabolic potential of uncultured microorganisms [9–12], the approach has some limitations. For example, it is difficult to connect mobile genetic elements such as plasmids and phages to metagenome-assembled genomes (MAGs) [13]. Generating MAGs from heterogeneous or low abundance populations is also challenging [14, 15]. Single-cell sequencing, in contrast, does not share these same limitations [5], and the approach has provided insights into microbial dark matter [4, 7], experimentally linked phages to their hosts [16, 17], and dissected natural populations [13, 18, 19]. However, multiple displacement amplification (MDA)—the predominant single-cell genome amplification method [20]— is limited by the poor uniformity and completeness of the genomes it produces [21]. Single-cell amplified genomes (SAGs) typically have genome completeness ≤40% [4].

Different variations on genome amplification chemistry [22–24] and sample processing strategies [25–31] have improved genome recovery in some situations, but an approach for consistently generating complete or nearly complete genomes from single microbial cells is still lacking. We recently developed primary template-directed amplification (PTA), which significantly improves amplified genome uniformity and variant calling in single human cells [32]. Here, we investigated whether PTA could also improve the quality of genomes recovered from single bacterial cells.

To benchmark PTA performance against the genome amplification chemistries commonly used in microbiome studies, we first sequenced the genomes of three bacterial isolate species: *Escherichia coli* (Gram-, GC% = 51%), *Pseudomonas putida* (Gram-, GC% = 62%), and *Bacillus subtilis* (Gram+, GC = 43%). Individual cells were sorted into 96-well plates using fluorescence activated cell sorting (FACS), and replicate plates were subjected to genome amplification using PTA, MDA, and whole-genome amplification (WGA)-X, a modified version of MDA that uses a more thermostable variant of phi29 polymerase [23] (Supplementary Table S1). Sequencing reads were mapped to reference genomes to measure coverage uniformity, and later assembled de novo using SPAdes [33]. All libraries were sub-sampled to 1 M reads (150 Mbp) prior to these analyses to ensure comparable sequencing effort among SAGs.

**Figure 1:**
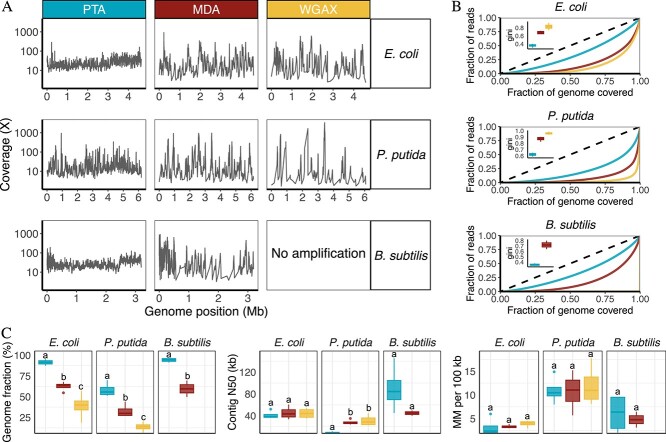
**Genome quality of *E. coli*, *P. putida*, and *B. subtilis* SAGs amplified using PTA (blue), MDA (red), and WGA-X (yellow). A)** Genome coverage of 500 bp windows from one representative replicate of each species amplified with each chemistry. WGA-X amplification reactions of *B. subtilis* failed and were not repeated. Refer to Supplementary Fig. 1 for genome coverage plots of all replicates. **B)** Uniformity of genome coverage illustrated by Lorenz curves and Gini coefficients. The dotted line represents the expected pattern of perfect uniform coverage, and solid lines illustrate the observed coverage for representative cells. **C)** Key summary statistics of de novo genome assemblies including completeness, contig N50, and the number of mismatches (MM) per 100 kb. The letters a, b, and c above the boxplots denote significance at the alpha 0.05 level. Sample sizes are n = 4 for all species and chemistries except for MDA amplified *B. subtilis,* which had n = 2. The boxplot dots represent outliers that are beyond the 1.5-fold interquartile range. Additional summary statistics are reported in Supplementary Fig. 2 and Supplementary Tables S2 and S3.

In every case, genome coverages from PTA reactions were significantly more uniform than MDA and WGA-X reactions based on Lorenz curves and Gini coefficients (Fig. 1 and Supplementary Table S2; *P* < .01 one way Analysis of Variance (ANOVA) and Tukey HSD for *E. coli* and *P. putida*; *P* < .01 one way t-test for *B. subtilis*). In addition, PTA amplification resulted in significantly greater genome completeness than did MDA and WGA-X for all three species (Fig. 1C and Supplementary Table S2; *P* < .01 one way ANOVA and Tukey HSD for *E. coli* and *P. putida*; *P* < .01 one way t-test for *B. subtilis*). For example, *B. subtilis* and *E. coli* SAGs assembled de novo had an average completeness of 94% and 91%, respectively, whereas genomes generated by MDA recovered only 60% and 62% on average (Supplementary Table S3). *P. putida* SAGs were less complete for all chemistries, but genomes generated by PTA were nearly 2-fold more complete than those generated by MDA and WGAX. *P. putida* genome completeness improved to 91% for PTA chemistry after increasing the number of input reads to an average of 4 M (600 Mbp); *P. putida* genomes amplified by MDA and WGAX were not sequenced deeply enough to assess completeness at this higher coverage level. PTA also showed similar fidelity to MDA and WGA-X when copying the genomes, e.g. no significant difference in genome mismatch rates per 100 kilobases among amplification chemistries (Fig. 1C and Supplementary Table S2; *P* > .05 one-way ANOVA). Overall, these results mirror the superior performance of PTA versus MDA and other genome amplification strategies observed previously using human cells [32].

**Figure 2:**
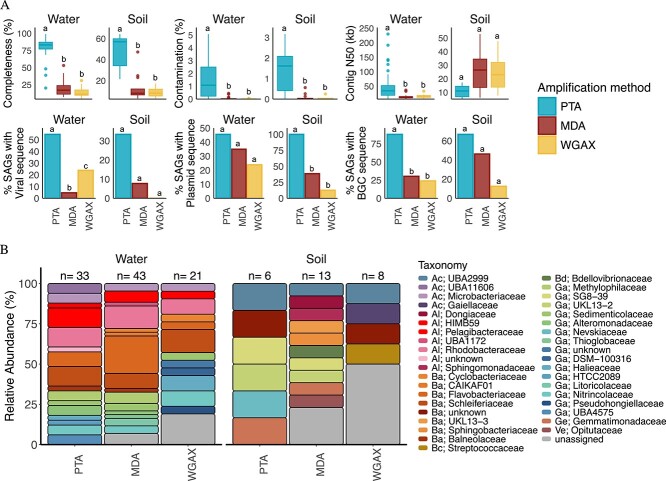
**Comparison of SAGs from aquatic and soil microbiomes amplified with PTA (blue), MDA (red), and WGA-X (yellow). A)** Estimated genome completeness and contamination using CheckM2 [34], contig N50, and the percentage of SAGs containing at least one predicted plasmid (> 5 kb), virus (> 5 kb), or BGC. The letters a, b, and c denote significance at the alpha 0.05 level. **B)** Family level taxonomic assignment of SAGs assembled from ≤20 Millon reads. Phylum / class abbreviations are as follows: Ac: Acidobacteria, Al: Alphaproteobacteria, Ba: Bacteroidota, Bd: Bdellovibrionota, Bc: Bacillota, Ga: Gammaproteobacteria, Ge: Gemmatimonadota, Ve: Verrucomicrobiota.

After performing these benchmarking experiments with bacterial isolates, we sought to determine if the improved performance of PTA could be extended to environmental samples. To accomplish this, we utilized the same comparison strategy to amplify and sequence single cells recovered by FACS from aquatic and soil samples. We again found that PTA resulted in significantly greater genome completeness than MDA and WGA-X (Fig. 2A and Supplementary Table S4; *P* < .01 one way ANOVA and Tukey HSD). For example, PTA reactions from aquatic samples had median genome completeness of 83%, while completeness from MDA and WGA-X reactions had medians of 17% and 11%, respectively (Fig. 2A and Supplementary Table S5). Deeper sequencing of MDA and WGA-X libraries to ~20 M reads (3 Gbp) increased median completeness estimates to 30% and 23%, respectively (Supplementary Table S5), but these genomes were still far less complete than those derived from PTA reactions (*P* < .01 one way ANOVA and Tukey HSD). Similar patterns were observed from a smaller soil microbiome dataset where PTA produced genomes with much greater completeness than MDA and WGA-X (Fig. 2A and Supplementary Table S4; *P* < .01 one way ANOVA and Tukey HSD). Additionally, a larger fraction of PTA genomes recovered from the aquatic system had virus and biosynthetic gene clusters (BGC) sequences, and a larger fraction of PTA genomes from soil had plasmid sequences (Fig. 2A and Supplementary Table S4; *P* < .05 Fisher’s exact test), presumably because PTA genomes were more complete than MDA and WGA-X genomes. Finally, phylogenetic analysis revealed successful PTA reactions on cells belonging to 20 families spread across 6 phyla (Fig. 2B), suggesting that PTA is amenable to a wide variety of microorganisms and produces substantially more near-complete genomes than standard amplification chemistries used in microbiome studies (Fig. 2 and Supplementary Fig. 3).

For single-cell genomes, overall genome quality is measured by a combination of completeness and contamination, with “high-quality” genomes defined as having >90% completeness and < 5% contamination [35]. Environmental genomes generated by PTA had a median contamination of 1.5% after applying an informatic decontamination procedure. Some contaminating sequences were found across SAGs and in no-template controls, suggesting that the PTA reagents contained trace levels of contaminating DNA. Single-cell whole genome amplification chemistries use short random primers to amplify a few femtograms of DNA, so even trace amounts of contaminating DNA can appear in assemblies. To decrease contaminating DNA, MDA, and WGA-X reagents underwent secondary treatment with UV prior to genome amplification [36]. PTA reagents underwent UV decontamination during manufacturing, but no secondary UV treatment of reagents or consumables was done prior to PTA reactions which may explain the higher contamination levels. Treating PTA reagents with an additional dose of UV may reduce contamination in future studies [28, 37]. Reducing reaction volume could also lower nonspecific synthesis from contaminant DNA and would lower reagent consumption and costs [28, 31].

In summary, our data shows that 78% of environmental SAGs produced with PTA were medium- or high-quality genome drafts (median completeness of 81% and 1.5% contamination), while >90% of the SAG produced with MDA and WGAX were classified as low-quality (median completeness <30%, and < 0.1% contamination) as defined by the Minimum Information about Single Amplified Genome standards [35] (Supplementary Fig. 3). Although SAGs from all chemistries were fragmented into many contigs–an on-going challenge for single-cell and metagenomic assembly–PTA often enabled recovery of nearly complete genomes from individual bacteria. We believe that these results set the stage for a renaissance in single-cell-based environmental genomics by offering a more comprehensive insight into the population structure of the microbial dark matter that accounts for a large fraction of the Earth’s biomass.

## Materials and methods

### Sample collection and processing

Fresh cultures of *E. coli* MG1655, *P. putida* KT2440, and *B. subtilis* pDR244 were grown overnight in LB at 37°C, then used immediately for cell sorting as described below. In addition, a 50 ml aquatic sample was collected from the surface waters of Mountain View Slough (latitude 37.432400, longitude −122.086632). The sample was vortexed for 15 s to release cells attached to sediment, filtered using a 15 μm cell strainer (pluriStrainer from pluriSelect, Germany) to remove large particles, and stored in 25% glycerol at −80°C until sorting. Finally, a soil microbiome sample was collected at Lawrence Berkeley National Laboratory (latitude 37.877382, longitude −122.250410). Approximately 5 g of soil was vortexed for 1 minute in 30 ml of water to release cells attached to sediment before centrifuging a 2 ml aliquot at 500 RCF for 5 minutes to pellet large particles. The supernatant was passed through a 5 μm filter and immediately used for cell sorting.

### Fluorescence activated cell sorting

Immediately before cell sorting, environmental bacteria and bacterial isolates were filtered through a 35 μm cell strainer to remove any remaining large particles and diluted to ~10^6^ cells/ml in filter-sterilized 1× PBS containing 1× SYBR-Green DNA stain (Thermofisher, USA). Individual cells were sorted using an Influx FACS machine (BD Biosciences) into LoBind 96-well plates (Eppendorf, Germany) containing either 3 μl of BioSkryb SL1-B Solution for PTA reactions or 1.2 μl of TE for MDA and WGA-X reactions. Plates were treated for 10 minutes in a UV crosslinker before sorting to remove any contaminating DNA. Cells were discriminated based on a combination of forward scatter characteristics and SYBR Green fluorescence. A single-cell sort mask with extra droplet discrimination was used to ensure only one cell was sorted into each well.

### Whole genome amplification

PTA was performed using the ResolveDNA Bacteria kit (BioSkryb Genomics, USA). Briefly, 3 μl of SL-B lysis reagent (BioSkryb Genomics, USA) was deposited in each well of a LoBind PCR plate (Eppendorf, Germany) prior to sorting. Plates containing sorted cells were film-sealed, briefly spun, mixed at 1400 rpm for 1 minute, and briefly spun again. The plates were then incubated at room temperature for 30 minutes to lyse the cells. Plates were stored at −80°C until ready to undergo whole genome amplification. PTA DNA amplification was carried as per the ResolveDNA Bacteria protocol (BioSkryb Genomics) unmonitored on a standard thermocycler for 12 hours at 30°C and in total reaction volumes of 20 μl, followed by 3 minutes at 65°C. Amplification kinetics were not monitored for PTA reactions, and all PTA reactions were selected for library creation and sequencing.

MDA was performed using Phi29 DNA Polymerase (Watchmaker Genomics, USA) as described previously [5] with 20 μl reaction volumes to match PTA reaction volumes. Similarly, 20 μl WGA-X [23] reactions were performed with EquiPhi29™ DNA Polymerase (Thermo Fisher). Alkaline cell lysis was performed after sorting and before genome amplification as described previously [5, 23]. In addition, a subset of MDA reactions received an additional Ready-Lyse (LGC Biosearch Technologies) lysozyme treatment of 50 U/μl for 15 minutes prior to alkaline lysis (Supplementary Tables S6 and S7). The kinetics of all MDA and WGA-X reactions were monitored by qPCR for background amplification. Reactions that amplified before no-template negative controls were considered successful and a subset was selected for sequencing library preparation. Supplementary Table S1 summarizes the number of total amplification reactions, successful reactions, and libraries sequenced.

Amplified DNA from PTA reactions and the selected MDA and WGA-X reactions were cleaned using SeraMagSelect beads at a 2× beads-to-sample ratio (Cytiva Life Sciences, USA) prior to sequencing library preparation.

### Library preparation and genome sequencing

Sequencing libraries were prepared from 10 to 100 ng of amplified DNA using the Nextera DNA flex library prep (Illumina, USA) using IDT for Illumina – Nextera DNA UD Indexes Sets A-D (Illumina, USA). Fragmentation times and amplification cycles were performed according to the ranges recommended by the manufacturer. Amplification reactions were cleaned using SPRI beads (Beckman Coulter, USA) at a 2× beads-to-sample ratio. Library concentrations and sizes were analyzed by TapeStation 2200 using D1000 ScreenTapes (Agilent, USA), and library concentration was determined using a Qubit fluorometer with DNA High Sensitivity reagents (Thermofisher, USA). Bacterial isolates and a subset of the aquatic environmental cells were sequenced on the NextSeq 2000 (Illumina), while the remaining libraries from aquatic and soil bacteria were sequenced on the Novaseq 6000 (Illumina) (Supplementary Tables S6 and S7). All libraries were sequenced using a 2×150bp read format.

### Read processing and genome assembly

Sequencing reads were filtered for quality using the rqc.filter2.sh script from BBTools Version 39.01 (https://bbtools.jgi.doe.gov) with following parameters: rna = f trimfragadapter = t qtrim = r trimq = 6 maxns = 1 maq = 10 minlen = 49 mlf = 0.33 phix = t removehuman = t removedog = t removecat = t removemouse = t khist = t removemicrobes = t sketch kapa = t clumpify = t rqcfilterdata=/clusterfs/jgi/groups/gentech/genome_analysis/ref/RQCFilterData barcodefilter = f trimpolyg = 5.

To generate assemblies from high and low levels of sequencing effort, each library was first subsampled to a maximum of 20 M and 1 M quality filtered reads (3 Gbp and 150 Mbp, respectively). Each subsampled library version was then normalized using bbtools.bbnorm with parameters: bits = 32 min = 2 target = 100 pigz unpigz ow = t. This normalization reduces the massive redundancy of reads from highly covered genome regions. Error correction was done on the normalized fastq using bbtools.tadpole with parameters: mode = correct pigz unpigz ow = t. Normalized reads were assembled using SPAdes v3.15.3 [33] using parameters: —phred-offset 33 -t 16 -m 64 —sc -k 25,55,95.

Assembled contigs were trimmed to remove 200 bp from each end, and contigs <2000 bp were removed to mitigate the impact of spurious short contigs [38–41].

### Genome quality assessment and taxonomic classification

The quality of SAGs derived from isolates was determined using QUAST version 5.2.0 [42]. Because sequencing effort varied substantially among bacterial isolate SAGs, assemblies made with 1 M reads were compared so that all replicates had equivalent sequencing depths. Genome coverage levels were determined by mapping each of the isolate SAGs against its corresponding reference genome: *E. coli* (IMG taxon ID: 2600254969), *P. putida* (IMG taxon ID: 2667527229) and *B. subtilis* (IMG taxon ID: 643886132). The bbmap parameters used in the analysis were bbmap.sh -Xmx100g fast = t 32bit = t. The resulting bam files were passed bedtools (v2.31.0) [43] to generate coverage files using the genomecov function. Lorenz curves and Gini coefficients were calculated from genomecov files using the R package gglorenz (v0.0.2). The Gini coefficient quantifies the observed deviation from perfect uniformity for each replicate cell, with smaller coefficients indicating more uniform coverage [44].

Environmental SAG assemblies were screened for contamination using a stepwise approach. First, we removed any human contigs. Next, we applied MAGpurify [45] in two sequential stages to remove contaminant contigs based on GC content and phylogenetic markers (stage 1) and tetranucleotide signatures (stage 2). Following the MAGpurify cleanup, we mapped reads generated from negative control reactions that lacked sorted cells and removed contigs with coverage >5X. Finally, we ran megablast against the NCBI non-redundant database and removed contigs with top hits to a set of organisms consistently found in the negative control reactions (Supplementary Table S8). Informatic decontamination reduced median contamination estimates for PTA SAGs from roughly 3% to 1.5% in genome versions assembled from 1 M reads (150 Mbp). Decontamination had little to no impact on MDA and WGA-X SAGs whose contamination levels were < 0.1% before treatment. Following contaminant removal, the quality of the environmental SAGs was assessed with CheckM2 (v1.0.1) [34] and MDMcleaner [46].

Assemblies from MDA and WGAX reactions were excluded from subsequent analyses if the total assembly size using all available reads was <0.2 Mbp and genome completeness was <2%. PTA assemblies entirely dominated by contaminant contigs and indistinguishable from assemblies of reagent-only, no-template controls were also excluded.

Statistical analysis of proportional results such as Gini coefficients, genome completeness, and genome contamination were performed on arcsine transformed data.

Taxonomic assignments of environmental SAGs were made with GTDB-tk (v2.3.2) [47]. SAGs derived from 20 M reads (3 Gbp)were used, when available, for taxonomic analysis because GTDB-tk struggled to make assignments to the less complete MDA and WGA-X genomes generated with 1 M reads (150 Mbp).

### Identification of viruses, plasmids, and biosynthetic gene clusters

Putative virus and plasmid contigs were identified by screening genomes with geNomad [48] using the end-to-end analysis parameter. Only hits >5 kb were included in downstream analyses. Of the viral contigs, 85% were predicted to be Caudoviricetes. Biosynthetic gene clusters were predicted using the JGI Secondary Metabolites Collaboratory pipeline which primarily uses antiSMASH v7.0 for prediction [49].

## Supplementary Material

ISMEComm_scMicrobe_PTA_Supplemental_Figures_ycae085

ISMEComm_scMicrobe_PTA_Supplemental_Tables_ycae085

## Data Availability

Raw sequencing reads were deposited in NCBI’s SRA (https://www.ncbi.nlm.nih.gov/sra), and annotated assemblies of environmental SAGs based on 1 M reads were deposited in the JGI’s Integrated Microbial Genomes and Microbiomes database (https://img.jgi.doe.gov/). Bioproject, biosample, and IMG genome IDs can be found in Supplementary Tables S6 and S7.
