# Supplementary material for: scMicrobe PTA: near complete genomes from single bacterial cells
Source: ISME Commun. 2024 Jul 12;4(1):ycae085. doi: 10.1093/ismeco/ycae085 (PMC11253033; doi:10.1093/ismeco/ycae085)
Supplement: ISMEComm_scMicrobe_PTA_Supplemental_Figures_ycae085 [file ismecomm_scmicrobe_pta_supplemental_figures_ycae085.pdf]

scMicrobe PTA: Near Complete Genomes from Single Bacterial Cells

Robert M Bowers[1 *], Veronica Gonzalez-Pena[2 *], Kartika Wardhani[2], Danielle Goudeau[1], Matthew James Blow[1], Daniel Udwary[1], David Klein[2], Albert C Vill[3], Ilana L Brito[3], Tanja Woyke[1], Rex Malmstrom[1 **], Charles Gawad[2,3 **]

* Robert Bowers and Veronica Gonzalez-Pena contributed equally
** Charles Gawad and Rex Malmstrom contributed equally

SUPPLEMENTAL FIGURES

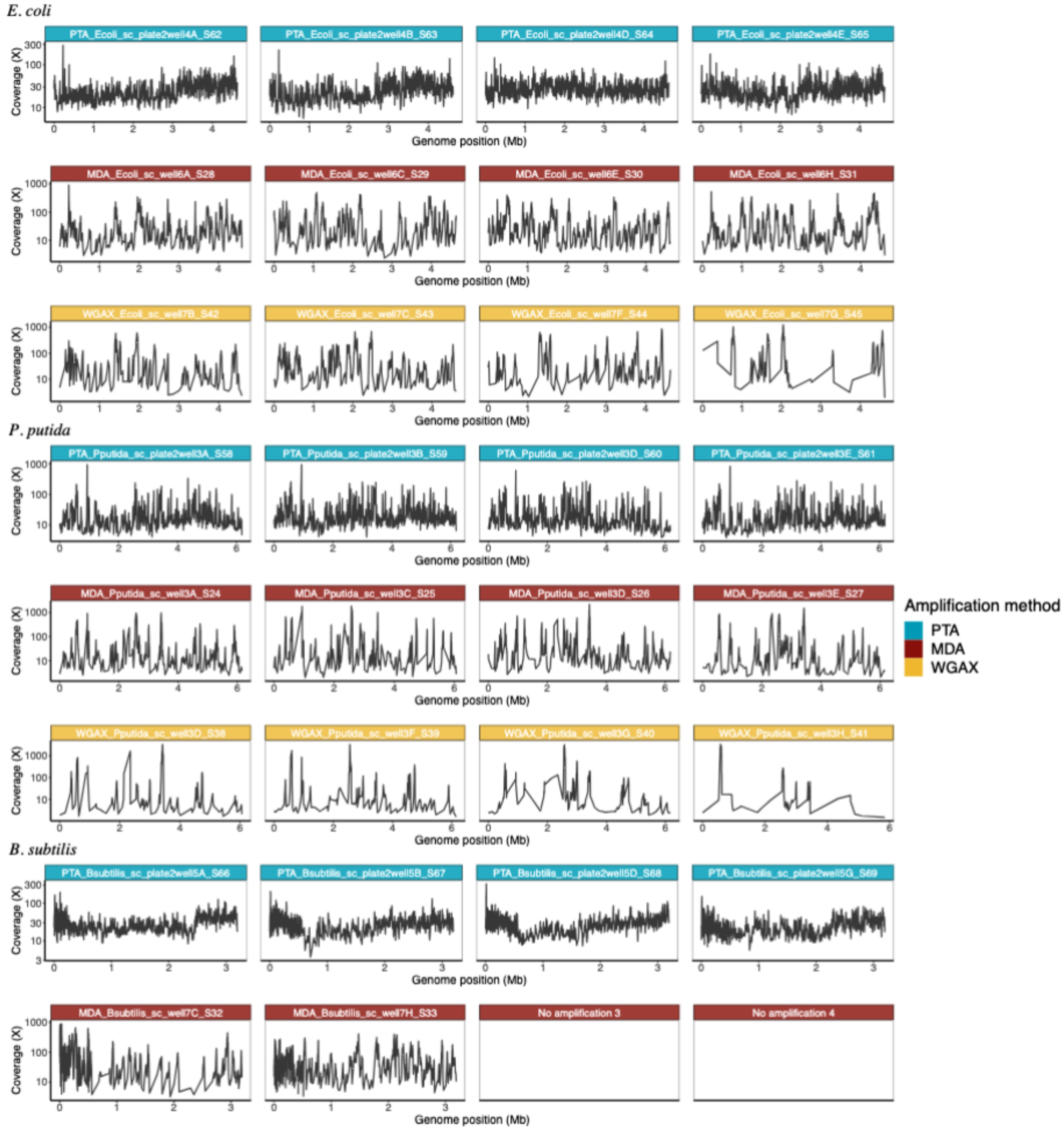

Supplementary Figure 1. Genome coverage of 500 bp windows of all replicates from each species amplified with each chemistry. WGA-X amplification reactions of *B. subtilis* failed and were not repeated, and only two MDA amplifications of *B. subtilis* were successful.

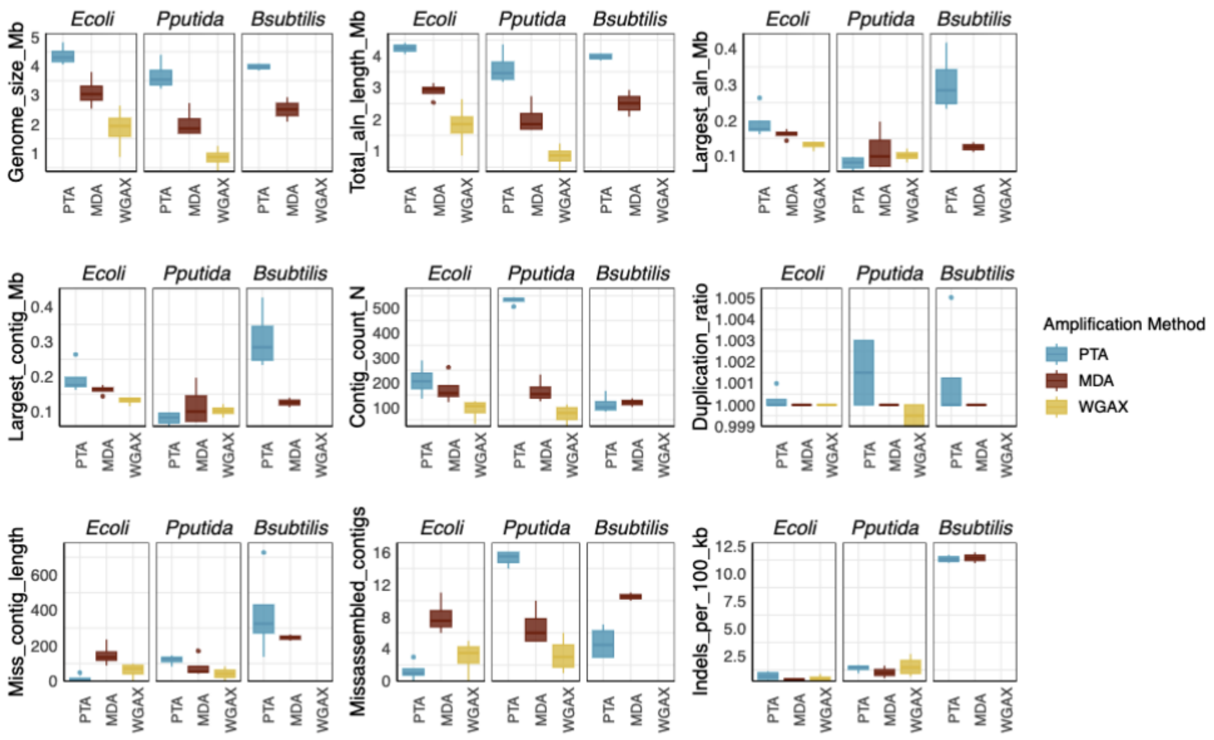

Supplementary Figure 2. Additional genome quality parameters not present in Fig. 1 of isolate single-cell genomes. Boxplots display the minimum, 25th percentile, median, 75th percentile and maximum values. The dots represent outliers that are beyond 1.5 * interquartile range.

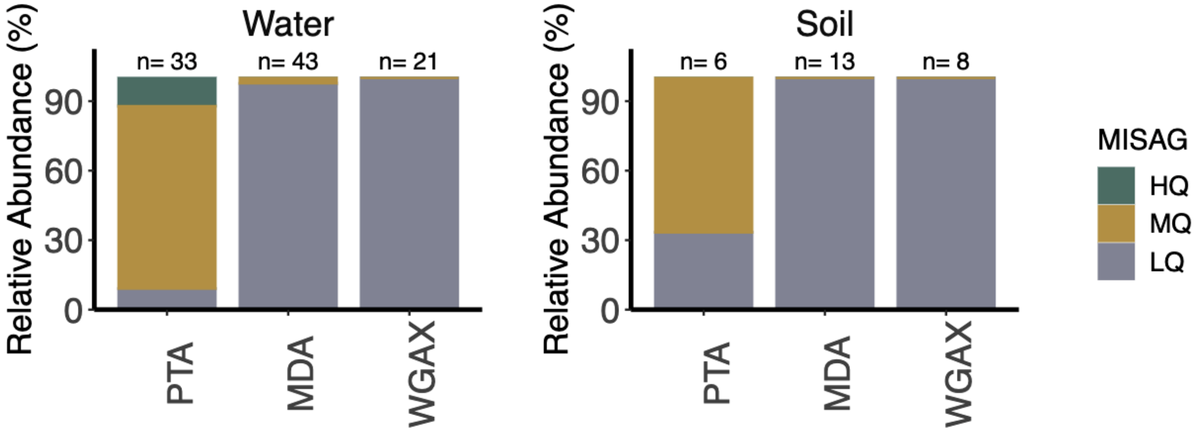

Supplementary Figure 3. Quality of environmental single cell genomes from aquatic and soil samples categorized per the minimum information MISAG standards criteria developed by the Genomic Standards Consortium (GSC). A High-quality (HQ) genome draft indicates that a SAG is > 90% complete with < 5% contamination, and the presence of the 23S, 16S and 5S rRNA genes and at least 18 tRNAs. Medium-quality (MQ) SAGs are genomes with completeness ≥50% and less than 10% contamination. All other SAGs (<50% complete with <10% contamination) are considered Low-quality (LQ) genome drafts [34]. The 4 genomes that made up the HQ fraction of the PTA aquatic samples satisfy these requirements, however the 16S rRNA genes were excluded from the final genomes as they were removed as a side-effect of the informatic decontamination procedure. This is a common problem when extracting MAGs from metagenomes [43], and for the same reasons, were removed after single cell decontamination likely due to variation in tetranucleotide frequencies.
